# Potential of *Eugenia brejoensis* (Mazine) Essential Oil in Combating Multidrug‐Resistant (MDR) *Acinetobacter baumannii* and *Pseudomonas aeruginosa*

**DOI:** 10.1002/cbdv.202501906

**Published:** 2025-11-05

**Authors:** Jonathan Mandú de Araújo, Amanda Vieira de Barros, Fábio Henrique Galdino Dos Santos, Weslley Felix De Oliveira, Daniela Maria do Amaral Ferraz Navarro, Márcia Vanusa da Silva, Bruno Oliveira de Veras, John Eversong Lucena de Vasconcelos, Henrique Douglas Melo Coutinho, Maria Betânia de Melo Oliveira, Maria Tereza dos Santos Correia

**Affiliations:** ^1^ Federal University of Pernambuco Recife Brazil; ^2^ CECAPE College Juazeiro do Norte Brazil; ^3^ Department of Biological Chemistry Regional University of Cariri – URCA Crato Brazil

**Keywords:** antimicrobial, biofilm, *Tenebrio molitor*, toxicity

## Abstract

The genus *Eugenia* has a long history of use in traditional medicine for treating various conditions, including infectious diseases, gastrointestinal disorders, and skin problems. Essential oils derived from *Eugenia* species are known for their medicinal properties and have been studied for their antimicrobial and bioactive potential. This study aimed to evaluate, through in vitro tests, the antibacterial, antibiofilm, and antibiotic‐modulating effects of *Eugenia brejoensis* essential oil (EOEb) against multidrug‐resistant (MDR) clinical isolates of *Acinetobacter baumannii* and *Pseudomonas aeruginosa* from COVID‐19 patients. In addition, we sought to analyze its toxicity and survival rates through in vivo tests in an invertebrate model using *Tenebrio molitor*. The EOEb was extracted via hydrodistillation and analyzed by gas chromatography–mass spectrometry (GC–MS) and flame ionization detector (FID). Clinical isolates of *A. baumannii* and *P. aeruginosa* were identified using matrix‐assisted laser desorption/ionization time‐of‐flight mass spectrometry (MALDI–TOF MS), and their resistance profiles were determined using the Vitek 2 system. The in vitro tests were conducted using the minimum inhibitory concentration (MIC) and minimum bactericidal concentration (MBC) were determined using microdilution and plating methods. Biofilm formation and inhibition assays were performed using crystal violet staining. Synergistic effects of EOEb with ciprofloxacin and gentamicin were evaluated using the checkerboard assay. The antibacterial effect of EOEb was tested in vivo using the *T. molitor* (mealworm) larvae model to assess toxicity and survival rates. The major constituents of EOEb were rosifoliol (16.47%), guaiol (12.69%), and (*E*)‐caryophyllene (11.97%). All bacteria exhibited an MDR profile. EOEb showed significant antibacterial activity against MDR strains, with MIC and MBC values ranging from 0.512 to 4.096 mg/mL. It also effectively inhibited biofilm formation at concentrations between 0.512 and 4.096 mg/mL. EOEb exhibited synergistic effects with ciprofloxacin against *A. baumannii* and with gentamicin against *P. aeruginosa*, as indicated by fractional inhibitory concentration (FIC) indices close to 0.5. The EOEb demonstrated low toxicity in the *T. molitor* model, with a survival rate of approximately 70%. The EOEb exhibits notable antimicrobial and biofilm‐inhibiting properties against MDR pathogens. Its low toxicity and synergistic effects with conventional antibiotics suggest its potential as a therapeutic alternative for combating antibiotic‐resistant infections.

## Graphical abstract

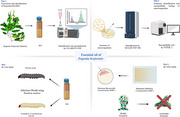

**Abbreviations**
EOEb: essential oil of *Eugenia brejoensis*
FIC: fractional inhibitory concentration
FID: flame ionization detector
GC–MS: gas chromatography–mass spectrometry
MALDI–TOF MS: matrix‐assisted laser desorption/ionization time‐of‐flight mass spectrometry
MBC: minimum bactericidal concentration
MDR: multidrug‐resistant
MIC: minimum inhibitory concentration

## 1 Introduction

Medicinal plants have been a cornerstone of human health for centuries, serving as vital therapeutic resources in both traditional and complementary medicine. In Brazil, particularly in the Northeast region, the use of medicinal plants is deeply embedded in cultural practices, with numerous species employed to treat infectious diseases, gastrointestinal disorders, skin conditions, and other ailments. This long‐standing tradition has catalyzed scientific interest in the bioactive properties of these plants, offering promising avenues for addressing hard‐to‐treat diseases and combating the growing challenge of antimicrobial resistance (AMR) [1, 2, 3].

Among the diverse flora with ethnopharmacological significance, plants from the *Eugenia* genus stand out due to their extensive use in traditional medicine. Indigenous and local communities across Brazil and other tropical regions have long relied on *Eugenia* species for their therapeutic benefits, including the treatment of diabetes, inflammatory diseases, and bacterial infections. The pharmacological properties of these plants are attributed to their rich composition of bioactive compounds, such as phenolics, flavonoids, and essential oils (EOs). These compounds not only underscore the medicinal value of *Eugenia* species but also position them as valuable resources for the development of novel pharmaceuticals, nutraceuticals, and functional foods. Bridging traditional knowledge with modern scientific research highlights the potential of these species in advancing health and wellness, while also emphasizing the importance of biodiversity conservation and sustainable utilization of these natural resources [4, 5].

The global health crisis posed by AMR has been further exacerbated by the excessive use of antibiotics during the COVID‐19 pandemic. Bacterial co‐infections were reported in up to 61% of COVID‐19 patients, particularly in low‐ and middle‐income countries like Brazil, and among individuals with comorbidities, leading to worsened clinical outcomes [5, 6, 7, 8]. The resistance profiles of these pathogens are often influenced by genetic alterations modulated by intrinsic and extrinsic factors, varying between different regions and countries, thus reflecting local epidemiological trends. Among the most concerning pathogens are *Acinetobacter baumannii* and *Pseudomonas aeruginosa*, which are frequently identified in hospital settings and are part of the ESKAPE group (*Enterococcus faecium*, *Staphylococcus aureus*, *Klebsiella pneumoniae*, *A. baumannii*, *P. aeruginosa*, and *Enterobacter* spp.). These pathogens are prioritized by the World Health Organization (WHO) for the development of new antimicrobial treatments due to their high resistance profiles and their significant role in hospital‐acquired infections [9, 10].

In this context, the search for novel compounds to combat AMR has become a critical focus of scientific research. Essential oils (EOs) extracted from plants have emerged as a promising alternative to conventional antibiotics. These complex mixtures of bioactive compounds, including terpenes, terpenoids, phenols, and aldehydes, exhibit broad‐spectrum antibacterial activity against a wide range of microorganisms, including MDR strains of *A. baumannii*, *Escherichia coli*, *P. aeruginosa*, and *S. aureus* [11, 12, 13, 14, 15]. Their multifaceted mechanisms of action, which often involve disrupting bacterial cell membranes, inhibiting biofilm formation, and modulating antibiotic resistance, make EOs particularly attractive as potential therapeutic agents.

This study focuses on the EO of *Eugenia brejoensis* (EOEb), a species with underexplored pharmacological potential. The research aims to chemically characterize EOEb, evaluate its efficacy in vitro against clinical isolates of *A. baumannii* and *P. aeruginosa* from COVID‐19 patients and assess its safety through in vivo toxicity tests using the *Tenebrio molitor* model.

## 2 Materials and Methods

### 2.1 Vegetal Material

Leaves of *E. brejoensis* were collected in April 2023 at the Catimbau National Park (8°34′47.73″ S; 37°14′55.36″ W) in the municipality of Buíque, Pernambuco, Brazil, a region of the Caatinga. The collected leaves were then washed with distilled water and dried at room temperature (25°C). Botanical identification of the specimen was carried out, and a voucher specimen was deposited in the Herbarium Professor Vasconcelos Sobrinho (PEUFR) of the Department of Biology at the Federal Rural University of Pernambuco (UFRPE), under the number 84.033.

### 2.2 EO Extraction

Approximately 500 g of dried leaves were ground and subjected to hydrodistillation (HD) for 4 h at 96°C using a Clevenger‐type apparatus. After HD, the EOEb obtained was dried with anhydrous sodium sulfate (Na_2_SO_4_), filtered, and weighed. The yield was calculated based on the obtained mass (m/m). Finally, EOEb was stored in amber glass bottles, refrigerated at −4°C until use.

### 2.3 Identification of EO Chemical Components

The EO was analyzed using gas chromatography coupled to mass spectrometry (GC–MS), using an Agilent Technologies (Palo Alto, CA, USA) Model 5975C, with an Agilent quadrupole detector and a DB‐5 apolar fused silica capillary column (30 m × 0.25 mm; film thickness 0.25 µm). For the injection, 1 µL of the sample (1000 ppm in hexane) was inserted into the system in Split mode (50:1), with the injector temperature set at 250°C. The oven was initially maintained at 40°C for 2 min, then increased to 230°C at a rate of 4°C/min and remained at 230°C for 5 min. Helium gas was used as the mobile phase (carrier gas), with a flow rate of 1 mL/min and a constant pressure of 7.0 psi. The mass detector source was set to 230°C and the quadrupole temperature was set to 150°C. The mass spectrum was generated at 70 eV (electron impact ionization), with a scan every 1 s, in the 35–350 *m*/*z* range. The retention indices were calculated by comparing the EO samples with a homologous series of hydrocarbons (C9–C32), using the equation of van den Dool and Kratz [16]. The mass spectra of the EO compounds were compared with the spectra in the GC/MS libraries (NIST08; WILEY7N; ESSENTIALOILS‐23P) and with data available in the literature, such as those by Adams (2007). Gas chromatography coupled to a flame ionization detector (GC–FID) was used for quantification, using a VB‐5 apolar column (Thermo Trace GC Ultra, 60 m × 0.25 mm; film thickness 0.25 µm), with nitrogen as the carrier gas and the same temperature conditions as the GC–MS. The injections were carried out in triplicate and the quantification of the chemical constituents was based on the relative areas of the peaks in the chromatogram.

### 2.4 Bacterial Isolates and Culture Condition

Ten clinical isolates were investigated in this study, comprising five isolates of *A. baumannii* and five isolates of *P. aeruginosa*. These isolates were obtained from hospitalized patients who tested positive for COVID‐19 at a public reference hospital in Recife, Pernambuco, Northeast Brazil. The collection and use of these isolates were approved by the Ethics Committee under protocol number CAA 64012522.5.0000.5208/CEP 5.848665.

#### 2.4.1 Taxonomic Identification Using Matrix‐Assisted Laser Desorption/Ionization Time‐of‐Flight

To confirm the taxonomy of the isolates, samples were cultivated in Brain Heart Infusion (BHI) agar medium (Kasvi) for 24 h and subsequently resuspended in deionized water, where proteins were extracted following the method described by Starostin et al. [17]. For mass spectrometry analysis, 1 µL of the protein extract was placed on a 96 MSP plate (Bruker Daltonics, Billerica, MA, USA) and air‐dried at room temperature. The matrix, α‐cyano‐4‐hydroxycinnamic acid (10 mg/mL) in 50% (v/v) acetonitrile and 0.3% (v/v) trifluoroacetic acid, was applied to the plate containing the samples for crystallization.

Spectra were acquired in positive linear mode (acceleration voltage of 20 kV and detection range of *m*/*z* 2000–20 000) using the Flex Control software, version 3.0 [18], and the MALDI–TOF Autoflex III mass spectrometer (Bruker Daltonics). The obtained spectra were compared with the MALDI Biotyper database (version 3.1). These results will generate a score that indicates the correlation between the experimentally acquired mass spectra and the reference profiles stored in the database. Accordingly, higher score values indicate a greater overlap between spectral profiles, thereby reflecting higher accuracy and reliability in taxonomic identification.

#### 2.4.2 Antibiotic Susceptibility of Isolates

The antibiotic susceptibility profiles of the clinical isolates were determined using the automated Vitek 2 Compact system (bioMérieux, Marcy‐l'Etoile, France) in the hospital laboratory, following the guidelines of the Brazilian Committee on Antimicrobial Susceptibility Testing [19]. The profiles were evaluated to classify the isolates as multidrug‐resistant (MDR) or extensively drug‐resistant (XDR) based on their resistance patterns. After characterization, the isolates were stored in 15% (v/v) glycerol in BHI medium (Kasvi) and maintained at −4°C for long‐term preservation. For experimental use, the isolates were reactivated by culturing on BHI agar (Kasvi) at 37°C.

This step ensured the accurate identification and characterization of the bacterial isolates, providing a reliable foundation for subsequent experiments evaluating the antimicrobial activity of EOEb.

### 2.5 Investigation of Antimicrobial, Antibiotic Modulating, and Antibiofilm Effects of EO

#### 2.5.1 Antibacterial Activity

The minimum inhibitory concentration (MIC) of EOEb was determined using the broth microdilution technique in sterile 96‐well flat‐bottom microplates, following the recommendations of the Clinical and Laboratory Standards Institute [20] with minor modifications. The microplates were filled with 100 µL of Mueller Hinton Broth (MHB) (Kasvi). Subsequently, 100 µL of EOEb previously diluted in 1% dimethyl sulfoxide (DMSO) were transferred to each well, followed by serial microdilution with 100 µL of the oil solution per column, resulting in a concentration gradient ranging from 4.096 to 0.128 mg/mL. Finally, 10 µL of the bacterial inoculum adjusted to a 0.5 McFarland standard concentration were distributed in the positive control and test wells. Wells containing only MHB were used as sterility controls, while those with MHB and the bacterial inoculum were used as growth controls (positive control). All tests were performed in triplicate, and the microplates were incubated at 37°C for 24 h.

Plate readings were taken using a spectrophotometer (Epoch) at 0 and 24 h, with a wavelength of 600 nm to obtain the optical density (OD). The determination of the concentration at which the EO was able to inhibit 50% of bacterial growth (MIC_50_) was based on the calculation described by Quave et al. [21]. After determining the MIC, 10 µL were removed from the wells where no growth was observed after 24 h of incubation (37°C) and then inoculated onto the surface of BHI agar plates (Kasvi). These were incubated for 24 h at 37°C, with the MBC being considered the lowest concentration at which no colonies formed under these conditions.

#### 2.5.2 Antibiotic Modulating Activity

The antibiotic modulating activity of EOEb was evaluated to determine its potential synergistic effects with conventional antibiotics. The concentrations of EOEb that exhibited inhibitory concentration 50 (MIC_50_) were selected for this analysis. Based on the resistance profiles provided by the Vitek 2 Compact system, the antibiotics gentamicin (NovaFarma) and ciprofloxacin (NovaFarma) were chosen for testing. The initial concentrations of the antibiotics were set at 8 µg/mL for gentamicin and 2 µg/mL for ciprofloxacin, based on the susceptibility profiles of the *A. baumannii* and *P. aeruginosa* isolates, respectively. In 96‐well microplates, serial dilutions of the antibiotics and EO were prepared in MHB. The bacterial inoculum, adjusted to a concentration of 0.5 McFarland standard, was added to each well, followed by incubation at 37°C for 24 h. Bacterial growth was assessed by measuring absorbance at 600 nm. The fractional inhibitory concentration index (ΣFICI) was calculated to quantify the interaction between the antimicrobial agents, using the formula ΣFICI = (MIC A + B/MIC A) + (MIC B + A/MIC B), where MIC A and MIC B represent the MICs of the antibiotics and EO, respectively, in combination and individually. The interactions were classified based on the following criteria: synergistic (FICI ≤ 0.75), additive (0.76 < FICI ≤ 1.0), indifferent (1 < FICI < 4), and antagonistic (FICI ≥ 4). Synergistic activity was considered when the combination inhibited ≥ 50% of bacterial growth compared to the MIC_50_.

#### 2.5.3 Biofilm Formation

Evaluation of the biofilm‐forming potential of the strains was determined using the crystal violet (CV) method [22] with some modifications. Essentially, in a polystyrene 96‐well microplate, 160 µL of culture medium (BHI), 20 µL of distilled water, and 20 µL of adjusted bacterial inoculum (1.5 × 10^8^ CFU/mL) were added to the wells. For sterility control, the bacterial inoculum was replaced with distilled water. After 24 h of incubation at 35 ± 2°C, the wells were washed three times with saline solution (NaCl 0.9%) to remove planktonic cells, followed by incubation at 55°C for biofilm fixation. Subsequently, 200 µL of CV was added to the wells for 15 min. The wells were then washed with distilled water and eluted with 100% ethanol to obtain the OD reading at a wavelength of 570 nm. Based on the absorbance readings (OD_570_), the mean absorbance values of each sample (OD_s_) compared to the sterility control (OD_c_) were determined. The samples were classified as strong biofilm formers (4× OD_c_ < OD_s_), moderate biofilm formers (2× OD_c_ < OD_s_ ≤ 4× OD_c_), and weak biofilm formers (OD_c_ < OD_s_ ≤ 2× OD_c_). Isolates with absorbance values equal to or less than the control were classified as non‐biofilm producers.

#### 2.5.4 Antibiofilm Formation Evaluation

For the evaluation of antibiofilm formation (biofilm inhibition), the parameters described by Trentin et al. [23] were used with some modifications. Briefly, 100 µL of the inhibitory concentrations 50 (MIC_50_) and sub‐inhibitory concentrations were added to a microdilution plate along with 10 µL of the bacterial suspension and 100 µL of BHI broth. The plate was incubated for 24 h at 37°C. After incubation, the content was removed, and the plate was washed three times with 0.9% saline solution. For biofilm fixation, the plate was incubated at 55°C for approximately 1 h. Subsequently, the biofilm was stained with 0.4% CV for 15 min, washed three times with 0.9% saline solution, and eluted with absolute ethanol for 30 min. The OD was measured at 570 nm and compared with the OD of the bacterial growth control. The percentage of viable cells was calculated using the following equation: $Cell\;viability=100x\left(OD_{570}\left(Sample\right)/OD_{570}\left(Control\right)\right).$

#### 2.5.5 Infection Model Using *T. molitor*

##### 2.5.5.1 Toxicity Assay

The toxicity of EOEb was evaluated using *T. molitor* larvae as an in vivo model. Larvae weighing approximately 100 mg each were randomly distributed into three experimental groups (*n* = 10 per group): a negative control group treated with 0.9% saline solution, a positive control group treated with 100% DMSO, and two treatment groups receiving EOEb diluted in 1% DMSO at concentrations of 2.048 mg/mL and 4.096 mg/mL. Using a Hamilton syringe equipped with a 26‐gauge needle, 5 µL of the respective solutions were injected into the caudal region of the larvae. The larvae were then incubated in the dark at 25°C for 5 days. Mortality was assessed daily, with larvae considered dead if they showed no movement in response to touch. Survival data were analyzed using Kaplan–Meier survival curves, and differences between groups were statistically evaluated using the log rank test. This assay provided critical information on the safety and toxicity profile of EOEb, ensuring its suitability for further in vivo antibacterial testing.

##### 2.5.5.2 Evaluation of Antibacterial Activity

The antibacterial activity of EOEb was further assessed using *T. molitor* larvae infected with *A. baumannii* or *P. aeruginosa*. Larvae weighing approximately 100 mg each were randomly distributed into groups of 10. A bacterial suspension (0.5 McFarland standard) of either *A. baumannii* or *P. aeruginosa* was injected into the hemocoel of the larvae using a Hamilton syringe with a 26‐gauge needle. The injection was performed by piercing the second or third visible sternite above the legs on the ventral side. Infected larvae were then treated with EOEb at a concentration of 1.024 mg/mL, which was determined based on previous in vitro results. Control groups included larvae injected with 0.9% saline solution to assess mortality due to handling or mechanical injury, as well as a toxicity control group treated with 100% DMSO. The larvae were kept in Petri dishes at 25°C in the dark for up to 5 days, and their viability was assessed daily. Mortality rates were recorded and compared between treated and control groups to evaluate the efficacy of EOEb in reducing bacterial infection and improving larval survival. This in vivo model provided valuable insights into the antibacterial potential of EOEb against MDR pathogens, complementing the in vitro findings and supporting its potential as a therapeutic agent.

### 2.6 Statistical Analysis

The antibacterial activity values were represented as percentage means ± standard deviation for each bacterial strain and were performed in triplicate. Statistically significant differences were analyzed using one‐way ANOVA followed by Tukey's test, with *p* ≤ 0.05 considered significant (GraphPad Prism 8 software).

## 3 Results and Discussion

### 3.1 Extraction and Chemical Composition of EO

The HD of *E. brejoensis* leaves yielded 0.54% of EO (EOEb). Chemical characterization using GC–MS and GC–FID identified 49 compounds, representing 96.57% of the total oil composition (Table 1).

**TABLE 1:** Chemical composition of *Eugenia brejoensis* Mazine (EOEb) by GC–MS and GC–FID.

Identity	RI determined	RI literature	(%) Composition	Standard deviation (%)
α‐Thujene	922	924	*trace* ^a^	—
α‐Pinene	928	932	1.00	0.06
Sabinene	968	963	0.37	0.02
β‐Pinene	969	974	0.30	0.03
Myrcene	987	988	0.47	0.02
α‐Terpinene	1011	1014	0.34	0.02
*p*‐Cymene	1018	1020	1.64	0.08
1,8‐Cineole	1024	1026	9.16	0.35
*(Z)‐*β‐Ocimene	1042	1032	*trace*	—
γ‐Terpinene	1051	1054	*trace*	—
Terpinolene	1078	1086	*trace*	—
Linalool	1090	1095	*trace*	—
Terpinen‐4‐ol	1171	1174	0.92	0.02
α‐Terpineol	1185	1186	1.01	0.02
Isoledene	1370	1374	*trace*	—
α‐Copaene	1373	1374	*trace*	—
β‐Bourbonene	1382	1387	*trace*	—
β‐Elemene	1389	1389	1.38	0.05
α‐Gurjunene	1407	1409	0.50	0.01
**(*E*)‐Caryophyllene**	1418	1417	**11.97**	0.31
β‐Gurjunene	1425	1431	*trace*	—
Aromadendrene	1436	1439	*trace*	—
α‐Humulene	1451	1452	1.45	0.03
(*E*)‐β‐Farnesene	1455	1454	trace	—
Allo‐Aromadendrene	1458	1458	0.41	0.01
γ‐Muurolene	1474	1478	*trace*	—
Germacrene D	1478	1484	*trace*	—
Bicyclogermacrene	1495	1500	4.04	0.09
Germacrene A	1503	1508	*trace*	—
γ‐Cadinene	1512	1513	*trace*	—
δ‐Cadinene	1521	1522	*trace*	—
α‐Calacorene	1541	1544	*trace*	—
Elemol	1550	1548	9.69	0.05
Germacrene B	1555	1559	0.21	0.00
β‐Calacorene	1562	1564	0.01	0.01
Spathulenol	1577	1577	1.26	0.05
Caryophyllene oxide	1582	1582	0.94	0.06
Globulol	1591	1590	*trace*	—
**Guaiol**	1602	1600	**12.69**	0.16
**Rosifoliol**	1612	1600	**16.47**	0.26
10‐*epi*‐γ‐Eudesmol	1620	1622	0.18	0.02
γ‐Eudesmol	1633	1630	5.45	0.04
β‐Eudesmol	1652	1649	6.92	0.56
α‐Eudesmol	1655	1652	4.76	0.62
Bulnesol	1668	1670	2.98	0.11
Cadalene	1673	1675	0.05	0.01
Eudesm‐7(11)‐en‐4‐ol	1693	1700	*trace*	—
Carissone	1926	1926	*trace*	—
Kaurene	2038	2042	*trace*	—
Total identified (%)			**96.57**	

^a^ Trace: < 0.01% of oil content.

The compounds were categorized into monoterpenes (26.53%), sesquiterpenes (71.43%), and a diterpene (2.04%). The major constituents identified were rosifoliol (16.47%), guaiol (12.69%), and (*E*)‐caryophyllene (11.97%), as detailed in Table 1. These findings align with previous studies on EOs from other species of the *Eugenia* genus, which have also reported the presence of sesquiterpenes and monoterpenes as major components [24, 25, 26, 27, 28]. In addition, the chemical profile of EOEb is consistent with earlier reports on *E. brejoensis*, which identified compounds such as δ‐cadinene (22.6%), β‐caryophyllene (14.4%), and α‐muurolol (9.34%) [1], as well as (*E*)‐caryophyllene, cadinene, and epi‐α‐muurolol [29]. Other studies have also reported similar major constituents, including δ‐cadinene (15.88%), *trans*‐caryophyllene (9.77%), and α‐muurolol (9.42%) [30], and β‐(*E*)‐caryophyllene (31.0%), δ‐cadinene (20.0%), and bicyclogermacrene (12.0%) [31].

A notable finding in this study was the identification of rosifoliol as the major compound in EOEb, which has not been previously reported for the *Eugenia* genus or *E. brejoensis* species. Rosifoliol, or 2‐(4a,8‐dimethyl‐3,4,5,6,7,8‐hexahydro‐2*H*‐naphthalen‐2‐yl) propan‐2‐ol according to the International Union of Pure and Applied Chemistry (IUPAC), has been identified in other plant species such as *Heliotropium bacciferum* Forssk. [32], *Helichrysum italicum* (Roth) G. Don [33], and *Glycosmis pentaphylla* (Retz.) DC. [34]. The presence of rosifoliol in EOEb highlights the unique chemical profile of this EO and underscores its potential as a source of novel bioactive compounds.

The qualitative and quantitative variations in the chemical composition of EOEb compared to previous studies can be attributed to factors such as edaphoclimatic conditions (e.g., soil type, climate, and altitude) and extraction conditions (e.g., drying method, extraction time, and temperature) [35, 36, 37].

Furthermore, this is the first report of the EO composition of leaves of *E. brejoensis* collected during the peak rainy months in the Catimbau National Park (April–June). Until now, our research group had only described the composition of this oil for collections carried out during the dry season [1, 31]. (*E*)‐Caryophyllene consistently appeared among the major constituents in all samples. Guaiol, identified as a minor constituent in the sample of Silva et al. [1], was detected as a major constituent in our sample collected during the period of extreme rainfall, alongside rosifoliol. This can be explained by the pronounced differences in precipitation, temperature, and soil conditions between the dry and rainy seasons, and their considerable influence on the production of secondary metabolites, such as EOs [38].

The detailed characterization of EOEb's chemical composition is crucial for understanding its pharmacological potential and guiding future research into its applications. The presence of compounds like rosifoliol, guaiol, and (*E*)‐caryophyllene, which are known for their bioactive properties, suggests that EOEb could serve as a valuable source of natural products for the development of new therapeutic agents. This study contributes to the growing body of knowledge on the *Eugenia* genus and highlights the importance of exploring the chemical diversity of plant species for drug discovery and development.

### 3.2 Identification and Susceptibility Profiling of Bacterial Isolates

The taxonomic identification of the bacterial isolates was confirmed using the automated Vitek 2 (Compact Biomerieux) system and further validated by matrix‐assisted laser desorption/ionization time‐of‐flight mass spectrometry (MALDI–TOF MS). The MALDI–TOF MS analysis yielded scores ranging from 1.9 to 2.31, indicating high compatibility and reliability in species identification. The identification and resistance profiles of *P. aeruginosa* strains are detailed in Table 2, while the profiles of *A. baumannii* isolates, previously characterized by Silva et al. [5], are also described in this study.

**TABLE 2:** Identification number, infection site, bacterial isolates identification by Vitek 2 and MALDI–TOF MS systems, MALDI–TOF score, resistance profile and resistance classification for *Acinetobacter baumannii* and *Pseudomonas aeruginosa* isolates.

ID	Infection site	Identification (Vitek 2 and MALDI–TOF MS)	Score Value MALDI–TOF MS	Antibiotic resistance profile	Classification of resistance	Reference
01	Catheter tip	*A. baumannii*	1.90	AMI/GEM/IMI/MER/LEV	MDR	Silva et al. [5]
02	Urine	*A. baumannii*	2.19	AMI/GEM/IMI/MER/LEV	MDR	
03	Urine	*A. baumannii*	2.23	AMI/GEM/IMI/MER/LEV	MDR	
04	Blood	*A. baumannii*	2.21	AMI/GEM/IMI/MER/LEV	MDR	
05	Blood	*A. baumannii*	2.12	AMI/GEM/IMI/MER/LEV	MDR	
01	Urine	*P. aeruginosa*	2.18	AMI/CEF/CAZ/IMI/MER/LEV/CIP	MDR	Author (2025)
02	Tracheal secretion	*P. aeruginosa*	2.00	CIP/LEV/IMI/PIP‐TAZ/MER	MDR	
03	Urine	*P. aeruginosa*	2.31	CIP/LEV/IMI/PIP‐TAZ/MER	MDR	
04	Tracheal secretion	*P. aeruginosa*	2.04	CIP/LEV/IMI/PIP‐TAZ/MER	MDR	
05	Wound secretion	*P. aeruginosa*	2.21	CIP/LEV/IMI/PIP‐TAZ/MER	MDR	

Abbreviations: AMI, amikacin (aminoglycosides); CAZ, ceftazidime (third generation cephalosporin); CEF, cefepime (fourth generation cephalosporin); CIP, ciprofloxacin (fluoroquinolone); GEM, gentamicin (aminoglycosides); ID, strain identification code; IMI, imipenem (carbapenem β‐lactam); LEV, levofloxacin (fluoroquinolone); MDR, multidrug‐resistant; MER, meropenem (carbapenem β‐lactam); PIP‐TAZ, piperacillin–tazobactam (penicillin + β‐lactamase inhibitor).

In this study, the isolates exhibited a MDR profile, consistent with the global prevalence of *A. baumannii* and *P. aeruginosa* as significant etiological agents of healthcare‐associated infections (HAIs). The increasing prevalence of these pathogens is linked to multiple resistance genes, posing a significant challenge due to the limited therapeutic options available [39, 40, 41].

*A. baumannii* is particularly concerning due to its high adaptability and resistance to adverse conditions. The global spread of carbapenem‐resistant *A. baumannii* has been documented in regions such as Asia, the Americas, and the Mediterranean, including Southern Europe, the Middle East, and North Africa [40]. In Brazil, the situation is equally alarming, with the *A. baumannii* complex exhibiting one of the highest carbapenem resistance rates, reaching approximately 80% [42].

During the COVID‐19 pandemic (2020–2021), there was a notable increase in unfavorable outcomes, such as higher mortality rates among patients co‐infected with *A. baumannii* and COVID‐19 [43]. These outcomes were likely exacerbated by factors such as overcrowded hospital wards, a shortage of trained infection control specialists, and reduced laboratory capacity to identify and control the spread of MDR microorganisms. These conditions created an environment conducive to the increased dissemination of resistant pathogens during and after the pandemic [44].

Similarly, infections caused by *P. aeruginosa* saw a significant rise during the pandemic. This increase has been attributed to factors such as the widespread use of corticosteroids and other immunomodulators, as well as the frequent use of invasive medical devices like mechanical ventilators and venous catheters [45, 46]. The indiscriminate use of antibiotics during this period further aggravated the problem of antibiotic resistance [45, 47], a trend reflected in this study, as all isolates were classified as MDR.

The findings of this study underscore the critical need for new therapeutic strategies to combat MDR pathogens like *A. baumannii* and *P. aeruginosa*. The high prevalence of resistance, particularly to carbapenems, highlights the urgency of developing alternative treatments, such as the use of natural products like EOEb, which has shown promising antimicrobial and biofilm‐inhibiting properties in this study.

### 3.3 Antibacterial Activity and Modulating Effect of Antibiotics

The antimicrobial activity of EOEb was evaluated against MDR strains of *A. baumannii* and *P. aeruginosa*. For *A. baumannii*, the MIC (MIC_50_) of EOEb ranged from 1.024 to 2.048 mg/mL, while the minimum bactericidal concentration (MBC) ranged from 1.024 to 4.096 mg/mL (Table 3). These results demonstrate the potential of EOEb to inhibit the growth of MDR *A. baumannii* at relatively low concentrations.

**TABLE 3:** Antibacterial activity and modulation effect of essential oil of *Eugenia brejoensis* (EOEb) with gentamicin against *Acinetobacter baumannii*.

ID	Strains	EOEb (mg/mL)		Modulatory activity		
		MIC_50_	MBC	EOEb (mg/mL)^a^	GEN (µg/mL)^b^	FICI
01	*A. baumannii*	1.024	2.048	1.024	0.125	2 (I)
02	*A. baumannii*	1.024	2.048	1.024	>8	2 (I)
03	*A. baumannii*	2.048	4.096	2.048	4	1.2 (I)
04	*A. baumannii*	1.024	4.096	0.512	0.250	0.52 (S)
05	*A. baumannii*	2.048	4.096	2.048	>8	2 (I)

Abbreviations: A, additive; FICI, fractional inhibitory concentration index; GEN, gentamicin; I, indifferent;

ID, strain identification code; MBC, minimum bactericidal concentration to kill 99.9% of microorganisms; MIC_50_, minimum inhibitory concentration to inhibit 50% of bacterial growth; S, synergistic.

^a^ EOEb concentration in modulation.

^b^ Antibiotic concentration in modulation;

To further explore the potential of EOEb, a modulatory activity study was conducted using gentamicin, an antibiotic commonly used to treat *A. baumannii* infections. The initial concentration of gentamicin was set at 8 µg/mL. The results revealed a synergistic effect (FICI = 0.52) for one isolate (isolate 04) of *A. baumannii*, while the other isolates showed an indifferent effect (1 < FICI < 4). Notably, the MIC_50_ of gentamicin for isolate 04 was significantly reduced to 0.125 µg/mL, compared to the control value of >8 µg/mL (Table 3). This reduction highlights the potential of EOEb to enhance the efficacy of gentamicin against MDR *A. baumannii*.

Previous studies have evaluated the antibacterial activity of *E. brejoensis* using ethanolic extracts of its leaves, reporting MIC and MBC values ranging from 3.12 to 50 mg/mL for various bacterial strains, including *S. aureus*, *Bacillus subtilis*, *E. coli*, *K. pneumoniae*, *Salmonella enteritidis*, and *P. aeruginosa* [48]. In addition, the EO has demonstrated larvicidal activity against *Aedes aegypti* (LC_50_ = 215 mg/mL) [1] and antibacterial activity against *Streptococcus mutans* (MIC = 62.5 µg/mL) [4]. The findings of this study contribute novel data by demonstrating the efficacy of EOEb against clinical isolates of *A. baumannii*, with inhibitory concentrations comparable to those reported for other EOs, such as *Origanum vulgare* L. [49].

The synergistic effect observed between EOEb and gentamicin aligns with previous studies that have demonstrated the potential of EOs to enhance the activity of conventional antibiotics. For example, Saleh et al. [50] reported synergistic effects when combining EOs from *Cinnamomum verum*, *Syzygium aromaticum*, *Thymus vulgaris*, and *Carum carvi* with imipenem against MDR *A. baumannii*. These findings underscore the potential of EOs as adjuvants to reduce antibiotic concentrations and combat resistance.

In contrast, the application of EOEb to other *A. baumannii* isolates did not show a significant modulatory effect, likely due to the intrinsic resistance of these strains, which is often associated with their high biofilm‐forming capacity and the presence of an outer membrane that limits the penetration of hydrophobic compounds. This highlights the complexity of bacterial resistance mechanisms and the need for tailored therapeutic strategies.

The study also evaluated the activity of EOEb against MDR isolates of *P. aeruginosa*. The MIC_50_ values ranged from 0.512 to 4.096 mg/mL across the five tested isolates. Furthermore, EOEb demonstrated a modulatory effect on the activity of ciprofloxacin, showing an additive effect for one isolate (isolate 01) and a synergistic effect for the others (Table 4). This is the first study to report the activity of EOEb against *P. aeruginosa*, and the results are consistent with previous findings on the antibacterial potential of EOs from other plant species, such as *Citrus aurantium*, *Curcuma longa*, and *Plectranthus amboinicus* [51].

**TABLE 4:** Antibacterial activity and modulation effect of essential oil of *Eugenia brejoensis* (EOEb) with ciprofloxacin against *Pseudomonas aeruginosa*.

ID	Strains	EOEb (mg/mL)		Modulatory activity		
		MIC_50_	MBC	EOEb (mg/mL)^a^	CIP (µg/mL)^b^	FICI
01	*P. aeruginosa*	1.024	2.048	1.024	0.03125	1 (A)
02	*P. aeruginosa*	2.048	4.096	0.512	0.0625	0.75 (S)
03	*P. aeruginosa*	4.096	>4.096	2.048	0.250	0.56 (S)
04	*P. aeruginosa*	4.096	>4.096	2.048	0.0625	0.52 (S)
05	*P. aeruginosa*	0.512	1.024	0.256	0.03125	0.53 (S)

Abbreviations: A, additive; CIP, ciprofloxacin; FICI, fractional inhibitory concentration index; I, indifferent; ID, strain identification code; MBC, minimum bactericidal concentration to kill 99.9% of microorganisms; MIC_50_, minimum inhibitory concentration to inhibit 50% of bacterial growth; S, synergistic.

^a^ EOEb concentration in modulation.

^b^ Antibiotic concentration in modulation.

The synergistic and additive effects observed between EOEb and ciprofloxacin against *P. aeruginosa* isolates highlight the potential of combining EOs with antibiotics to reduce the required concentrations of both agents. This approach not only enhances antimicrobial efficacy but also minimizes toxicity and delays the development of resistance. Similar findings have been reported for other EOs, such as *Cinnamomum zeylanicum*, which exhibited MIC and MBC values ranging from 0.625 to 5 mg/mL against MDR *P. aeruginosa* [52].

The high concentrations of EOs required to inhibit *P. aeruginosa* growth, often reaching up to 100 mg/mL [53], underscore the challenges posed by this pathogen's resistance mechanisms. However, the combinatorial approach of using EOs with antibiotics offers a promising strategy to overcome these challenges. By reducing the necessary concentration of both agents, this approach addresses the dual issues of resistance and toxicity, as demonstrated in this study and supported by previous research [54, 55, 56].

In conclusion, the findings of this study highlight the antibacterial and antibiotic‐modulating potential of EOEb against MDR strains of *A. baumannii* and *P. aeruginosa*. The synergistic and additive effects observed with conventional antibiotics suggest that EOEb could serve as a valuable adjuvant in the treatment of infections caused by these highly resistant pathogens. Further research is needed to explore the mechanisms underlying these interactions and to evaluate the clinical applicability of EOEb in combination therapies.

### 3.4 Biofilm Formation and Anti‐Biofilm Activity

**FIGURE 1:**
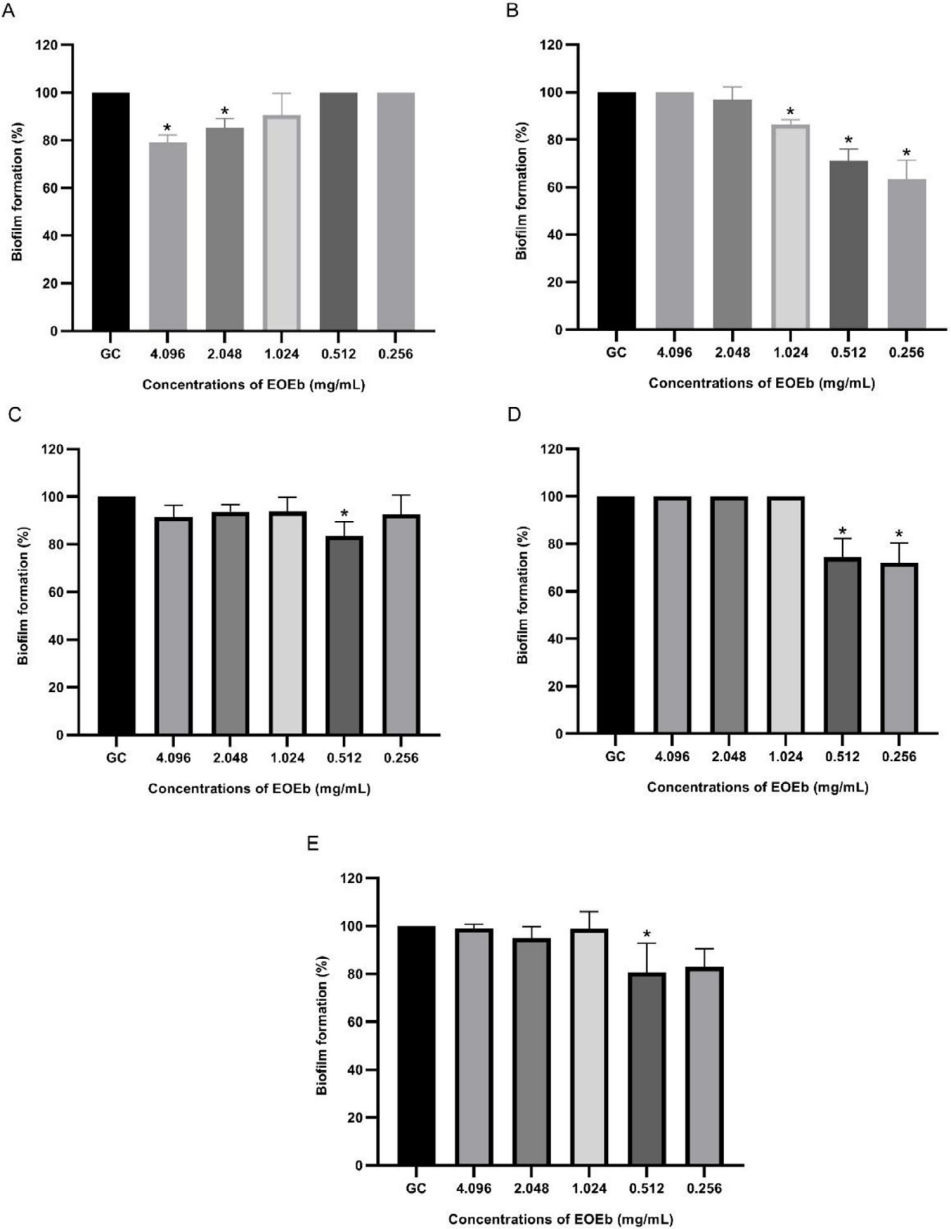
Anti‐biofilm activity of *Eugenia brejoensis* essential oil (EOEb) on *Acinetobacter baumannii* isolates (A–E). The experiment was performed in triplicate. Statistical significance was *p < 0.05 compared to the growth control. Cg: growth control; A–E: *A. baumannii* strains ID 1–5, respectively.

The biofilm formation assay revealed that all five *A. baumannii* isolates were strong biofilm producers (4 × OD_c_ < OD_s_). The anti‐biofilm potential of EOEb was evaluated, and the results are depicted in Figure 1. EOEb demonstrated significant anti‐biofilm activity against the tested *A. baumannii* strains, with Tukey's test confirming a statistically significant difference between the control and treatment groups (*p* < 0.05).

The concentrations of EOEb required to inhibit biofilm formation in *A. baumannii* isolates ranged from 0.256 to 4.096 mg/mL. Specifically, 0.256 mg/mL inhibited biofilm formation in isolates Ab2 (36.4 ± 7.9%) and Ab4 (28 ± 8.4%), while 0.512 mg/mL was effective against isolates Ab3 (16.4 ± 5.9) and Ab5 (19.3 ± 12.1%), as well as reinforcing inhibition in Ab2 (29 ± 4.9%) and Ab4 (25.7 ± 7.9%). These results highlight the significant anti‐biofilm activity of EOEb at relatively low concentrations. Higher concentrations, such as 1.024 mg/mL, also showed inhibitory effects, particularly for isolate Ab2 (13.6%), while 2.048 and 4.096 mg/mL inhibited biofilm growth in isolate Ab1 by 14.7 ± 3.8% and 20.9 ± 3.1%, respectively.

Interestingly, the anti‐biofilm activity of EOEb exhibited a nonlinear dose–response effect, resembling an inverted U‐shaped response. This phenomenon, known as hormesis, suggests that the anti‐biofilm activity is most pronounced at intermediate concentrations, with lower or higher concentrations showing reduced efficacy [57, 58]. This behavior underscores the complexity of interactions between EOEb and bacterial biofilms, which may involve multiple mechanisms of action.

The strong biofilm‐forming capacity of *A. baumannii* isolates is consistent with their high levels of AMR, which is often mediated by virulence factors such as the *ompA* and *bap* genes [59]. However, biofilm formation is a multifactorial process, and there is evidence suggesting an inverse relationship between antibiotic resistance and biofilm production capacity [60]. This complexity highlights the challenges in treating biofilm‐associated infections, particularly in clinical settings where *A. baumannii* is a leading cause of HAIs [59, 61].

The anti‐biofilm activity of EOEb aligns with findings from other studies evaluating EOs from different plant species. For example, *C. zeylanicum* EO inhibited biofilm formation in MDR *A. baumannii* strains at concentrations ranging from 0.25 to 0.5 mg/mL [62], like the results observed in this study for isolates Ab2, Ab3, Ab4, and Ab5. Similarly, Ouslimani et al. [63] reported anti‐biofilm concentrations of 0.4–52 mg/mL for EOs from 20 plant species against MDR, XDR, and pandrug‐resistant (PDR) *A. baumannii* strains, further corroborating the high resistance and virulence of these pathogens.

**FIGURE 2:**
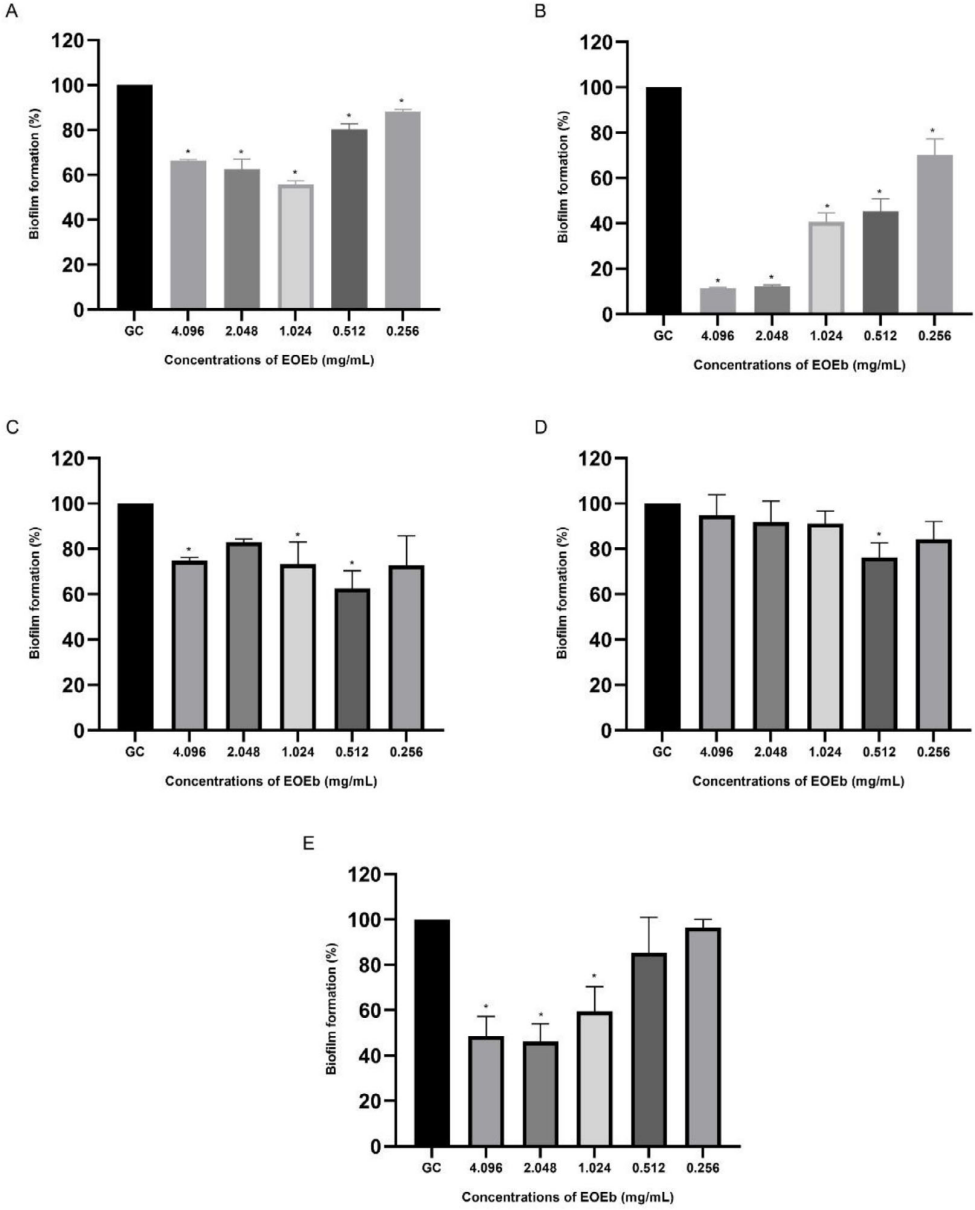
Anti‐biofilm activity of *Eugenia brejoensis* essential oil (EOEb) on *Pseudomonas aeruginosa* isolates (A–E). The experiment was performed in triplicate. Statistical significance was *p < 0.05 compared to the growth control. Cg: growth control; A–E: *P. aeruginosa* strains ID 1–5, respectively.

For *P. aeruginosa*, the biofilm formation assay confirmed that all isolates were strong biofilm producers (4 × OD_c_ < OD_s_). The anti‐biofilm potential of EOEb against these isolates is illustrated in Figure 2. The strong biofilm‐forming capacity of *P. aeruginosa* is consistent with literature reports, particularly in the context of SARS‐CoV‐2 co‐infections, where the virus creates a conducive environment for bacterial adaptation and chronic infection [28, 64].

EOEb demonstrated significant anti‐biofilm activity against *P. aeruginosa* isolates, with inhibition observed at concentrations ranging from 0.256 to 4.096 mg/mL. Isolate Pa02 was the most sensitive, showing 87.9 ± 0.7% and 88.6 ± 0.4% inhibition at 2.048 mg/mL and 4.096 mg/mL, respectively. In contrast, isolate Pa04 was the most resistant, with only 23.7 ± 6.3% inhibition at 0.512 mg/mL. These findings align with previous studies suggesting that *P. aeruginosa* strains from SARS‐CoV‐2 patients exhibit high biofilm‐forming capacity and resistance to conventional antibiotics [65].

This study is the first to evaluate the anti‐biofilm activity of EOEb against *P. aeruginosa*, and the results are consistent with those reported for EOs from other plant species. For example, *Salvia officinalis* and *Ocimum basilicum* EOs inhibited biofilm formation in MDR *P. aeruginosa* at concentrations ranging from 5 to 20 mg/mL [66]. Similarly, Sena et al. [67] reported biofilm inhibition concentrations of 0.04–5 mg/mL for EOs from nine plant species when combined with antibiotics, further supporting the potential of EOEb as an anti‐biofilm agent.

The anti‐biofilm activity of EOEb may be attributed to its high hydrophobicity, which can interfere with bacterial adhesion and biofilm formation [68]. In addition, the interaction of EOEb components with signal receptors, inhibition of signaling molecule biosynthesis, degradation of quorum‐sensing molecules, and down‐regulation of biofilm‐related genes may also play a role [69]. These mechanisms highlight the multifaceted nature of EOEb's anti‐biofilm activity, which warrants further investigation.

In conclusion, the findings of this study demonstrate the significant anti‐biofilm potential of EOEb against MDR strains of *A. baumannii* and *P. aeruginosa*. The ability of EOEb to inhibit biofilm formation at relatively low concentrations suggests its potential as a natural alternative for preventing and treating biofilm‐associated infections. By targeting the initial stages of biofilm formation, EOEb may help reduce bacterial colonization and infection development, offering a promising strategy to combat the growing challenge of AMR. Further research is needed to elucidate the underlying mechanisms and explore the clinical applicability of EOEb in biofilm‐related therapies.

### 3.5 Infection Model Using *T. molitor*

To evaluate the toxicity and antibacterial efficacy of EOEb, an infection model using *T. molitor* (mealworm) larvae was employed. This model is increasingly utilized in biomedical research due to its rapid breeding, low cost, ease of handling, and immunological similarity to mammals [70, 71].

**FIGURE 3:**
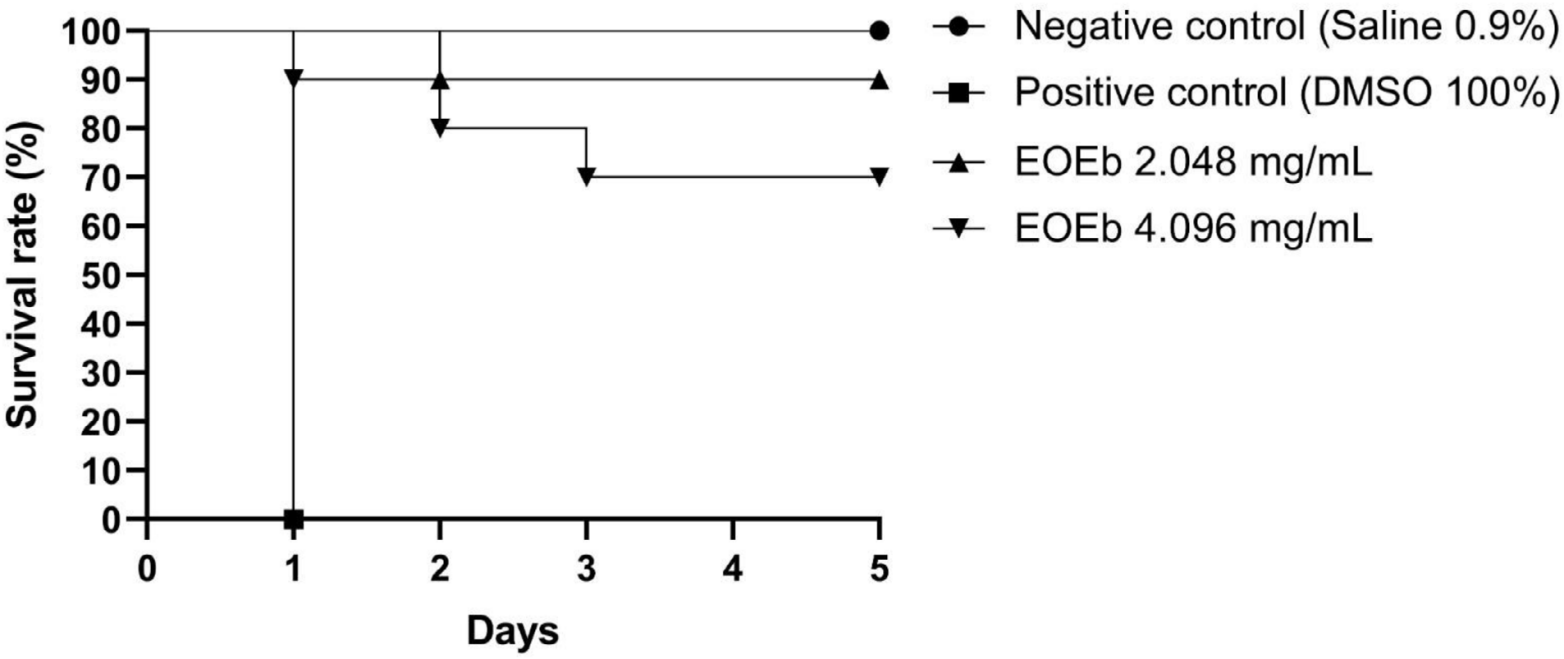
Survival impact of *Eugenia brejoensis* essential oil (EOEb) on *Tenebrio molitor* larvae.

The toxicity of EOEb was assessed at the two highest concentrations tested in vitro: 2.048 and 4.096 mg/mL. The survival curves for the larvae are illustrated in Figure 3. The results demonstrated that EOEb was well‐tolerated by the larvae, with 4.096 mg/mL resulting in 30% mortality and 2.048 mg/mL causing only 10% mortality, compared to the negative control (0.9% saline), which showed no mortality. In contrast, larvae treated with 100% DMSO exhibited 100% mortality within the first day post‐administration, highlighting the low toxicity of EOEb even at high concentrations.

The *T. molitor* model has been widely used to evaluate the toxicity of various compounds, including antiviral drugs such as acyclovir and molnupiravir, which showed no toxicity at doses above 2 mg/mL [72]. While *T. molitor* assays do not replace mammalian studies, they serve as a valuable intermediate tool between in vitro experiments and more complex in vivo models. This approach allows for the preliminary identification of potentially toxic compounds and provides initial insights into pharmacokinetic parameters, contributing to a more efficient and safer transition to mammalian studies. Comparisons with other studies further support the low toxicity of EOEb. For example, a 2% concentration of *Cedrus deodara* EO caused 50% mortality in *T. molitor* larvae [73], while Pereira et al. [4] demonstrated that EOEb concentrations of 62.5 and 625 µg/mL did not induce toxic effects in *T. molitor*. These findings reinforce the safety profile of EOEb, even at higher concentrations, and underscore its potential as a therapeutic agent.

**FIGURE 4:**
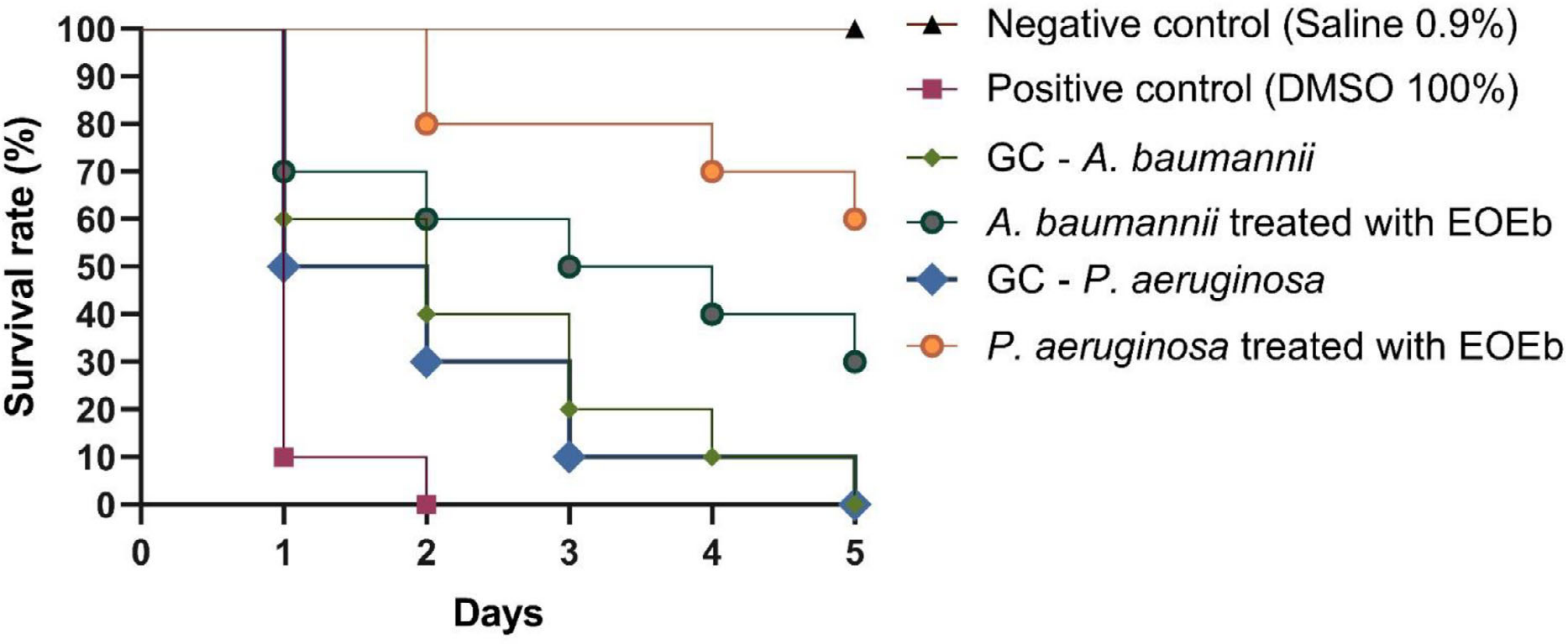
Survival analysis of *Tenebrio molitor* larvae infected with *Acinetobacter baumannii* (ID 01) and *Pseudomonas aeruginosa* (ID 01) and treated with *Eugenia brejoensis* essential oil (EOEb). CG, growth control.

To validate the antibacterial efficacy of EOEb, an infection assay was conducted using *T. molitor* larvae inoculated with *A. baumannii* (ID 01) and *P. aeruginosa* (ID 01). A standardized concentration of 1.024 mg/mL of EOEb was used, as this concentration was common to the MIC_50_ values for both isolates. The survival curves generated from the experimental data are presented in Figure 4. In the negative control group (0.9% saline), all larvae survived the experimental procedure, while the positive control (100% DMSO) resulted in 100% mortality within 2 days. For *A. baumannii* (ID 01), the infection control group exhibited 100% mortality by the end of the 5‐day evaluation period. In contrast, larvae treated with 1.024 mg/mL of EOEb showed a 30% survival rate, suggesting that EOEb was able to partially reverse the infection and improve larval survival despite the high resistance of the strain.

For *P. aeruginosa* (ID 01), the infection control group also showed 100% mortality after 5 days. However, treatment with EOEb resulted in a 60% survival rate, indicating a more pronounced antibacterial effect against this pathogen. This difference in efficacy may be attributed to the modulation of bacterial virulence factors or the stimulation of immune responses in the larvae, highlighting the potential of EOEb as a therapeutic agent.

The results of this study align with previous research investigating the efficacy of EOEb in alternative infection models. For example, Bezerra‐Filho et al. [2] demonstrated that EOEb increased the lifespan of *Caenorhabditis elegans* and *Galleria mellonella* infected with *S. aureus* (ATCC 29312). In the *G. mellonella* model, EOEb significantly reduced the severity of infection, as indicated by decreased melanin production, a marker of immune response and infection severity. These findings further support the potential of EOEb as an effective antibacterial agent in vivo.

The results of this study highlight the low toxicity and antibacterial efficacy of EOEb in the *T. molitor* infection model. The ability of EOEb to improve survival rates in larvae infected with highly resistant pathogens such as *A. baumannii* and *P. aeruginosa* underscores its potential as a therapeutic alternative for combating infections caused by these challenging pathogens.

The *T. molitor* model provides a cost‐effective and ethically viable platform for preliminary in vivo evaluations, bridging the gap between in vitro studies and mammalian experiments. However, further research is needed to elucidate the mechanisms of action underlying the antibacterial and immunomodulatory effects of EOEb, as well as to evaluate its efficacy and safety in more complex biological systems. In conclusion, the findings of this study demonstrate the potential of EOEb as a safe and effective therapeutic agent against MDR bacterial infections. Its low toxicity and ability to improve survival in an in vivo infection model highlights its promise for future development as a natural alternative to conventional antibiotics.

## 4 Conclusions

This study highlights the significant potential of EOEb as a natural antimicrobial agent against MDR strains of *A. baumannii* and *P. aeruginosa*, demonstrating potent antibacterial activity with MIC_50_ values ranging from 0.512 to 4.096 mg/mL, synergistic effects with antibiotics like gentamicin and ciprofloxacin, and effective biofilm inhibition at concentrations as low as 0.256 mg/mL. In the *T. molitor* infection model, EOEb exhibited low toxicity, with survival rates of 70% and 90% at concentrations of 4.096 and 2.048 mg/mL, respectively, and significantly improved survival rates in larvae infected with *A. baumannii* and *P. aeruginosa*. These findings underscore EOEb's potential as a natural alternative to conventional antibiotics, offering a promising strategy to combat MDR pathogens through its antimicrobial, anti‐biofilm, and low‐toxicity properties, while future research should focus on elucidating its mechanisms of action, evaluating its efficacy in mammalian models, and exploring its use as an adjuvant in combination therapies to address the global challenge of AMR.

## Author Contributions

**Jonathan Mandú de Araújo**: conceptualization, methodology, validation, writing – original draft, investigation, formal analysis, data curation, project administration. **Amanda Vieira de Barros**: methodology, investigation, formal analysis, writing – review and editing. **Fábio Henrique Galdino Dos Santos**: methodology. **Weslley Felix De Oliveira**: writing – review and editing. **Daniela Maria do Amaral Ferraz Navarro**: methodology. **Márcia Vanusa da Silva**: methodology. **Bruno Oliveira de Veras**: writing – review and editing, visualization. **Maria Betânia de Melo Oliveira**: writing – review and editing, supervision, project administration. **Maria Tereza dos Santos Correia**: supervision, funding acquisition.

## Conflicts of Interest

The authors declare no conflicts of interest.

## Data Availability

The data that support the findings of this study are available from the corresponding author upon reasonable request.
